# Identifying Malignant Pleural Effusion by A Cancer Ratio (Serum LDH: Pleural Fluid ADA Ratio)

**DOI:** 10.1007/s00408-015-9831-6

**Published:** 2015-12-17

**Authors:** Akash Verma, John Abisheganaden, R. W. Light

**Affiliations:** Department of Respiratory and Critical Care Medicine, Tan Tock Seng Hospital, 11 Jalan Tan Tock Seng, Singapore, 308433 Singapore; Pulmonary Disease Program, St. Thomas Hospital and Vanderbilt University, 4220 Harding Rd., Nashville, TN 37205 USA

**Keywords:** Cancer (lung), Pleural effusion, Lactate dehydrogenase, Adenosine deaminase, Biopsy

## Abstract

**Aim:**

We studied the diagnostic potential of serum lactate dehydrogenase (LDH) in malignant pleural effusion.

**Methods:**

Retrospective analysis of patients hospitalized with exudative pleural effusion in 2013.

**Results:**

Serum LDH and serum LDH: pleural fluid ADA ratio was significantly higher in cancer patients presenting with exudative pleural effusion. In multivariate logistic regression analysis, pleural fluid ADA was negatively correlated 0.62 (0.45–0.85, *p* = 0.003) with malignancy, whereas serum LDH 1.02 (1.0–1.03, *p* = 0.004) and serum LDH: pleural fluid ADA ratio 0.94 (0.99–1.0, *p* = 0.04) was correlated positively with malignant pleural effusion. For serum LDH: pleural fluid ADA ratio, a cut-off level of >20 showed sensitivity, specificity of 0.98 (95 % CI 0.92–0.99) and 0.94 (95 % CI 0.83–0.98), respectively. The positive likelihood ratio was 32.6 (95 % CI 10.7–99.6), while the negative likelihood ratio at this cut-off was 0.03 (95 % CI 0.01–0.15).

**Conclusion:**

Higher serum LDH and serum LDH: pleural fluid ADA ratio in patients presenting with exudative pleural effusion can distinguish between malignant and non-malignant effusion on the first day of hospitalization. The cut-off level for serum LDH: pleural fluid ADA ratio of >20 is highly predictive of malignancy in patients with exudative pleural effusion (whether lymphocytic or neutrophilic) with high sensitivity and specificity.

## Introduction

Exudative effusion is commonly seen in three conditions namely cancer, tuberculosis (TB) and parapneumonic effusion. Assessment and comparison of serum lactate dehydrogenase (LDH) and protein, with the pleural fluid LDH and protein based on Light`s criteria, to determine the exudative or transudative nature of the effusion is the first step in the management of pleural effusion [1–4]. Once an exudative effusion is identified, further work-up entails its biochemical analysis for cell count, glucose, pH, adenosine deaminase (ADA), cytology and TB culture. This is followed by pleural biopsy when the biochemical results are inconclusive.

Initial treatment decisions are based on changes in the biochemical markers, such as high levels of LDH, low levels of pH and glucose, and neutrophil predominance that aid in the diagnosis of pyogenic effusion (parapneumonic, empyema) and guide regarding the need for antibiotics, drainage or surgical decortication [5]. Similarly, a raised level of ADA helps to diagnose tubercular pleural effusion with the sensitivity and specificity of 0.92 (95 % confidence interval 0.90–0.93) and 0.90 (95 % confidence interval 0.89–0.91), respectively [6].

However, no reliable biochemical marker is available to aid the diagnosis of malignant pleural effusion. Often the low levels of ADA are used as a surrogate indicator of malignant effusion while waiting for the cytology result. This is compounded by the low yield of cytology which is only 50 % for malignant effusion [7, 8]. When negative, a closed or thoracoscopic pleural biopsy is indicated to establish the diagnosis of cancer, out of which the closed pleural biopsy adds only 8 % to the overall yield [9]. As a result, many times, the effusion remains undiagnosed in cases when the patient refuses the thoracoscopic biopsy or when it is unavailable. This impedes timely initiation of the treatment of lung cancer.

Serum lactate dehydrogenase is a ubiquitous cellular enzyme, which rises in response to tissue injury in a non-specific manner [10]. Consequently, elevated serum LDH is present in numerous clinical conditions, such as haemolysis, cancer, sepsis, human immunodeficiency virus infection and many others [10]. However, a very high and isolated serum LDH might be a marker of specific diagnostic groups. Its diagnostic and prognostic role has previously been reported mainly as a marker of poor outcome in sepsis and cancer patients [11–19]. The proposed explanation for its rise in cancer is the preferential use of glycolysis for energy, instead of oxidative phosphorylation by tumour cells, which is mediated by LDH [20, 21]. However, the diagnostic potential of this simple clinical biomarker for malignant pleural effusion has not been reported.

Since it is routinely done as the part of the well-established initial work-up of pleural effusion in all patients hospitalized for it, we did the current study to evaluate if its level on admission can also be utilized to discriminate between malignant, tubercular and parapneumonic effusions.

## Methods

We performed retrospective analysis of 163 patients hospitalized for the management of “exudative” pleural effusion in the year 2013. Patients with the discharge diagnosis of pleural effusion were searched using the ICD code. Those in whom pleural effusion was transudative were excluded from analysis. We collected data on the biomarkers, such as serum LDH, serum C-reactive protein (CRP) and the pleural fluid analysis results, done within 24 h of hospitalization. The confirmation of final diagnosis was based on pleural fluid cytology or pleural biopsy histology result in case of malignancy, acid fast bacilli growth on pleural fluid or pleural biopsy tissue in case of TB and growth of pyogenic organism on pleural fluid culture or resolution of infection with antibiotics in case of parapneumonic effusion. We analysed the serum LDH: pleural fluid ADA ratio as predictor of malignant pleural effusion and describe it as a “cancer ratio”. Institutional Review Board approval was obtained for this study with the waiver of consent (DSRB reference no. 2015/00488).

### Data Analysis

We used software (SPSS, version 17; SPSS, Chicago, Ill) for all statistical analyses. The results were compared using a Wilcoxon two-sample test or Fisher exact test. *P* values were two sided and considered indicative of a significant difference if <0.05.

## Results

Among 163 patients with exudative pleural effusion analysed, one hundred patients had malignant pleural effusion, out of which 95 had lung cancer, and the aetiology of the rest of the patients with malignant pleural effusion was as follows: ovarian cancer (*n* = 1), cervical cancer (*n* = 1), breast cancer (*n* = 1), malignant melanoma (*n* = 1) and mesothelioma (*n* = 1). Among the remaining 63 patients with benign aetiology, forty patients had tubercular effusion, 14 had parapneumonic effusion and nine were undiagnosed.

**Fig. 1:**
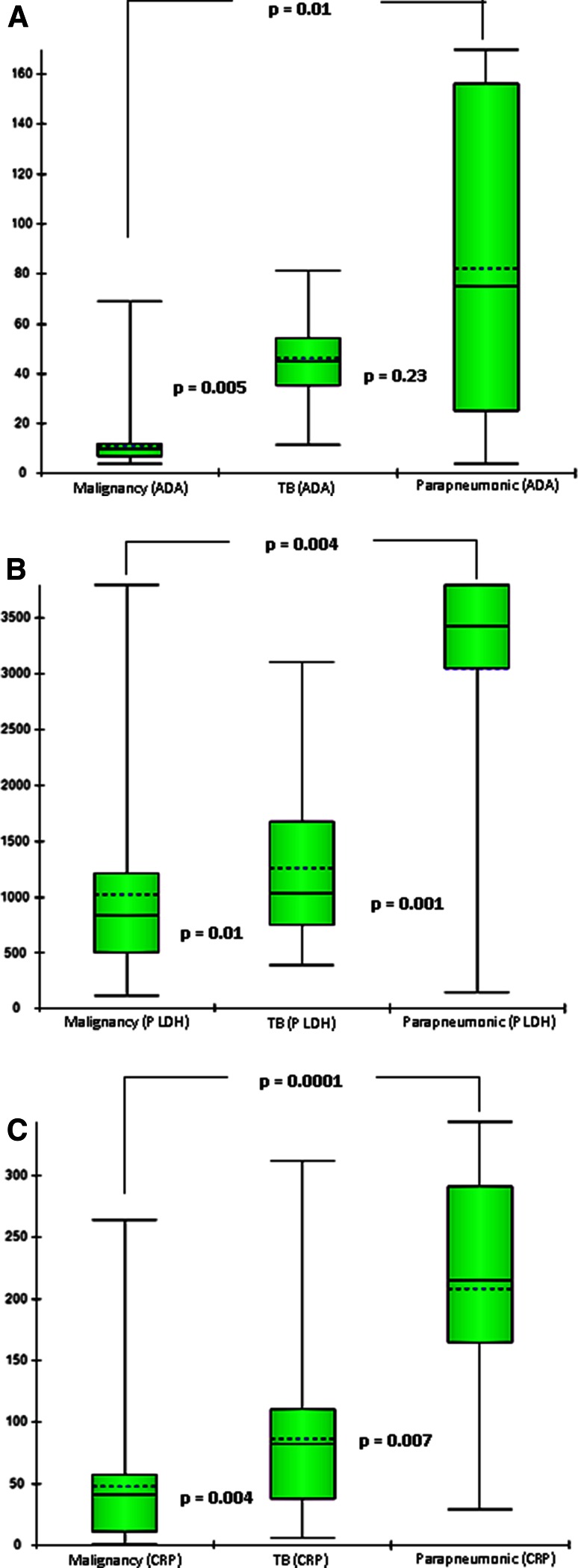
Comparison of pleural fluid ADA, pleural fluid LDH and serum CRP level between malignant, tubercular and parapneumonic pleural effusion. **a** Pleural fluid ADA was significantly lower in the malignant pleural effusion as compared to tubercular or parapneumonic effusion. **b** Pleural fluid LDH was significantly lower in the malignant pleural effusion as compared to tubercular & parapneumonic effusion, and it was highest in Parapneumonic effusion. **c** Serum CRP level was significantly lower in the malignant pleural effusion as compared to tubercular & parapneumonic effusion, and it was highest in parapneumonic effusion

**Fig. 2:**
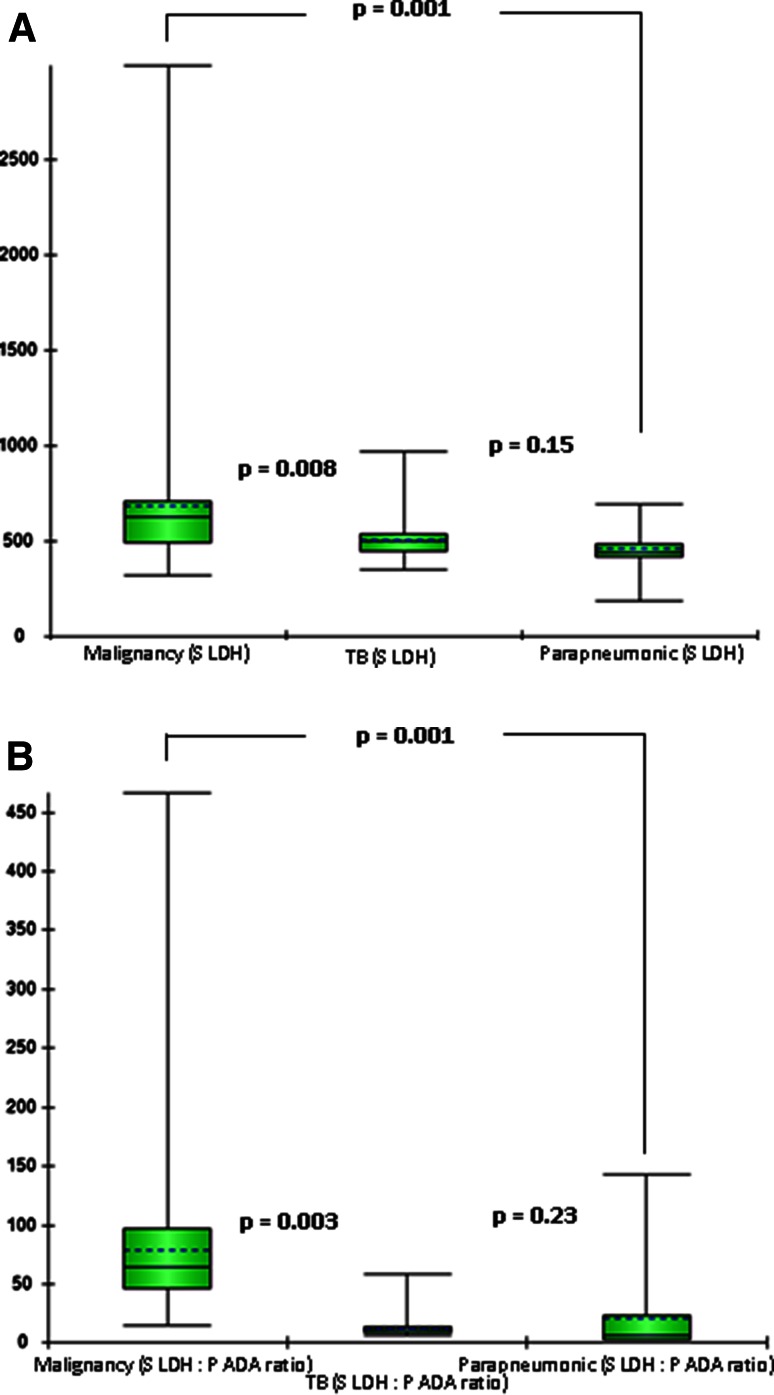
Comparison of serum LDH and serum LDH: pleural fluid ADA ratio between malignant, tubercular and parapneumonic pleural effusion. **a** Serum LDH was significantly higher in the malignant pleural effusion as compared to tubercular or parapneumonic effusion and it was similar between the latter two. **b** Serum LDH: pleural fluid ADA ratio was significantly higher in the malignant pleural effusion as compared to tubercular & parapneumonic effusion, and it was similar between the latter two

Univariate analysis showed biomarkers of systemic inflammation, such as serum CRP, and pleural inflammation, such as pleural fluid LDH to be raised in pleural effusion of infectious aetiology such as TB and parapneumonic effusion (Fig. 1). On the contrary, both these inflammatory markers were significantly lower in advanced lung cancer. Serum LDH on the other hand was raised to a significantly higher level in cancer patients discriminating between malignant and non-malignant exudative effusion (Fig. 2). When combined with pleural fluid ADA level, as serum LDH: pleural fluid ADA ratio, a further discriminating effect between malignant and non-malignant effusion is shown in Table 1.

**Table 1:** Univariate analysis of biomarkers of inflammation, LDH and ADA

	Cancer *N* = 100	Tuberculosis *N* = 40	Parapneumonic effusion *N* = 14	*P* value	*P* value	*P* value
	A	B	C	A&B	A&C	B&C
Pleural ADA	10 (4–69.3)	45.15 (11.7–81.4)	74.95 (4–170)	0.005	0.01	0.23
Pleural LDH	834.5 (117–3800)	1037 (395–3101)	3800 (142–3800)	0.01	0.004	0.001
Serum LDH	627 (320–2992)	509.5 (352–974)	439 (191–694)	0.008	0.001	0.15
Serum CRP	41.05 (1.2–263.6)	82.55 (6.3–311.6)	214.65 (29.1–343.7)	0.004	0.0001	0.007
Serum LDH: pleural ADA ratio	64.97 (14.86–467)	11.24 (6.08–58.29)	6.53 (2.56–143.25)	0.0003	0.0001	0.23
Pleural LDH: S LDH ratio	1.33 (0.19–5.56)	2.13 (0.58–7.97)	8.66 (0.25–9.74)	0.004	0.0004	0.001

Data presented as median (range)

*ADA* adenosine deaminase, *LDH* lactate dehydrogenase, *CRP* C-reactive protein

In multivariate logistic regression analysis, pleural fluid ADA was a negative, and serum LDH was a positive predictor of malignant pleural effusion shown in Table 2.

**Table 2:** Multivariate logistic regression analysis with malignancy as the outcome variable

Variable	Coefficient	Odds	P value
Pleural ADA	−0.4726	0.623 (0.45–0.85)	0.0031
Pleural LDH	−0.0041	0.995 (0.99–1.001)	0.1466
Serum LDH	0.0205	1.020 (1.00–1.03)	0.0041
Serum CRP	−0.0005	0.999 (0.98–1.01)	0.9603
Serum LDH: pleural ADA ratio	0.0586	0.943 (0.99–1.00)	0.0428
Pleural LDH: serum LDH ratio	2.4554	11.650 (1.00–135.48)	0.05

*ADA* adenosine deaminase, *LDH* lactate dehydrogenase, *CRP* C-reactive protein

### Serum LDH: Pleural Fluid ADA Ratio Cut-off Level

**Fig. 3:**
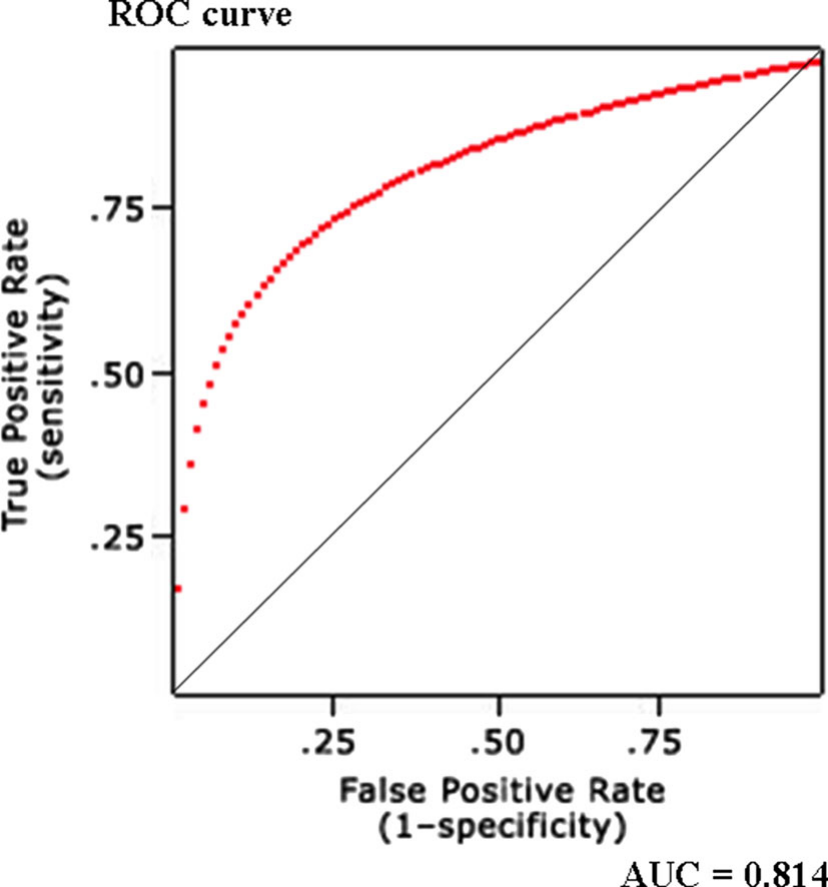
ROC curve for various cut-off levels of Serum LDH: pleural fluid ADA ratio in differentiating between malignant pleural effusion and effusion due to TB or infection

For serum LDH: pleural fluid ADA ratio, at the cut-off level of >20, the sensitivity and specificity were 0.98 and 0.94 respectively. The positive likelihood ratio (PLR) value was 32.6, while the negative likelihood ratio (NLR) at this cut-off was found to be 0.03 (Fig. 3). Table 3.

**Table 3:** Serum LDH: pleural fluid ADA—sensitivity and specificity at different cut-off level

Cut-off level	Sensitivity (95 % CI)	Specificity (95 % CI)	PPV (95 % CI)	NPV (95 % CI)	PLR (95 % CI)	NLR (95 % CI)
>10	1.0 (0.95–1)	0.44 (0.31–0.58)	0.76 (0.68–0.83)	1.0 (0.82–1)	3.3 (2.4–4.6)	0
>20	0.98 (0.92–0.99)	0.94 (0.83–0.98)	0.97 (0.90–0.99)	0.96 (0.85–0.99)	32.6 (10.7–99.6)	0.03 (0.01–0.15)
>30	0.89 (0.80–0.94)	0.94 (0.83–0.98)	0.96 (0.90–0.99)	0.82 (0.70–0.90)	30 (9.8–91.3)	0.21 (0.12–0.37)
>40	0.81 (0.71–0.87)	0.96 (0.86–0.99)	0.97 (0.90–0.99)	0.73 (0.61–0.82)	40.5 (10.2–159.3)	0.36 (0.24–0.54)
>60	0.53 (0.42–0.62)	0.98 (0.88–0.99)	0.98 (0.88–0.99)	0.53 (0.42–0.62)	53 (7.6–369.5)	0.88 (0.7–1.1)

*PPV* positive predictive value, *NPV* negative predictive value, *PLR* positive likelihood ratio, *NLR* negative likelihood ratio

## Discussion

Serum LDH and serum LDH: Pleural fluid ADA ratio is significantly higher in patients presenting with malignant pleural effusion and hence discriminate between malignant and non-malignant effusion. In particular, a cut-off level for serum LDH: pleural fluid ADA ratio of > 20 is highly predictive of malignancy in patients with exudative pleural effusion (whether lymphocytic or neutrophilic) with high sensitivity and specificity.

Serum lactate dehydrogenase is a ubiquitous cellular enzyme, which rises in response to tissue injury in a non-specific manner [10]. Consequently, elevated serum LDH is found in numerous clinical conditions [10]. However, a very high and isolated serum LDH might be a marker of specific diagnostic groups. Its diagnostic and prognostic role has previously been studied and reported as a poor prognostic marker in sepsis and cancer patients [11–19].

The proposed explanation for its rise in cancer is the preferential use of *glycolysis* for energy by tumour cells, instead of *oxidative phosphorylation*, a switch in the ATP generation pathway which is mediated by LDH [20, 21]. High rate of glycolysis is advantageous to growing cells because it is capable of producing ATP considerably faster than oxidative phosphorylation. Since growing cells have an enormous demand for ATP to fuel their growth, glycolysis is much better suited to meeting this demand [22]. Clinically, this property is utilized by positron emission tomography (PET) imaging of tumour uptake of 18F-2-deoxyglucose to visualize cancer. It is the enzyme LDH that mediates the reaction that permits the regeneration of NAD+, needed as an electron acceptor to maintain glycolysis [23]. However, its diagnostic potential as a biomarker for malignant pleural effusion has not been reported. Our findings of correlation between raised serum LDH and malignant pleural effusion is in keeping with previously reported correlation between serum LDH and cancer [24–26].

### Serum CRP Versus Serum LDH

Since serum LDH is known to rise in a non-specific manner in response to tissue injury, we analysed the well-established marker of systemic inflammation, i.e. CRP done within 24 h of hospitalization, and compared it with Serum LDH done within 24 h of hospitalization in patients presenting with exudative pleural effusion. Our results showed that CRP was higher in patients with infective effusions in keeping with its property as an *acute*-*phase reactant*, whereas serum LDH was higher in cancer patients. An increase in CRP level in lung cancer patients compared to healthy individuals has been described [27, 28]. However such comparison is not adequate for attributing raised CRP to cancer, as lung cancer patients may have concomitant inflammation from other sources such as cancer-related pulmonary infection. In our cohort of malignant pleural effusion, the CRP level was raised; however, it was lower in comparison to infective effusion. This finding argues in favour of the specific relationship of serum LDH with cancer (exudative pleural effusion of malignant aetiology), rather than serum CRP.

### Serum LDH: Pleural Fluid ADA Ratio (Cancer Ratio)

ADA is secreted by mononuclear cells, lymphocytes, neutrophils and red blood cells [29, 30]. It is of two types, ADA-1 and ADA-2, however, only total ADA is measured in the routine clinical practice. High levels correlate with infective conditions such as TB (ADA-2) and empyema (ADA-1) [31, 32]. In our cohort, the median ADA level was 45.15 (11.7–81.4) and 74.95 (4–170) in TB and parapneumonic effusions, respectively, in keeping with published literature.

ADA level is known to be low in malignant effusion. However, it is not appropriate to use these low levels to diagnose malignant effusion due to lack of biochemical relationship between them. Serum LDH, however, has been shown to be high in malignancies with the well-studied mechanism [24–26]. For this reason, we combined the two markers with negative and positive correlation with malignancy in an attempt to develop a predictor of malignant pleural effusion. This ratio was significantly higher in the malignant group, versus the TB and parapneumonic effusion group.

### Cut-off Level

Determining the cut-off value requires a compromise between sensitivity and specificity [33]. ADA is a reasonable tool for diagnosing TB and the recommended cut-offs are >35 or >40. The summary estimates for ADA in the diagnosis of tuberculous pleurisy in the meta-analysis reported sensitivity 0.92 (95 % confidence interval 0.90–0.93), specificity of 0.90 (95 % confidence interval 0.89–0.91), positive likelihood ratio 9.03 (95 % confidence interval 7.19–11.35) and NLR of 0.10 (95 % confidence interval 0.07–0.14) [6].

A highly sensitive test is good for screening. It will, however, have a tendency to give a greater number of false positive results. This may lead to a false alarm for cancer and mental agony. High specificity makes the test more definitive for the diagnosis. As the cytology is negative in 50 % of the patients, we focussed on high specificity with reasonable sensitivity. The cut-off of 20–30 gave us reasonable sensitivity and specificity; however, we chose >20 as the recommended cut-off as the NLR was 0.03 at this cut-off.

At the cut-off level of >20, the PLR value was 32.6 suggesting that patients with cancer have about 32 fold higher chance of having *cancer ratio* (Serum LDH: Pleural fluid ADA ratio) of >20 compared with patients without cancer. This high probability would be considered high enough to consider an effusion very likely to be malignant. On the other hand, NLR at this cut-off was found to be 0.03 which suggests that if the *cancer ratiois* <*20*, the probability that this patient has cancer is 3 %, which is low enough to make the diagnosis of cancer highly unlikely. These data suggest that a lower ratio (<20) can be used alone as a justification to consider a benign diagnosis such as TB or parapneumonic effusion. Additionally, the PLR and NLR for this “cancer ratio” were comparable with the ratios of ADA for TB.

The limitation of our study is its retrospective nature. Second, a few of the serum LDH samples were haemolysed. Haemolysis due to various reasons can cause the serum LDH to be falsely high. However, this is unlikely to have significant effect in our cohort as all three groups had an equal proportion of haemolysed samples. Third, we did not study the other causes of exudative effusions such as connective tissue diseases to validate these results in this group of patients. Fourth, most patients with malignant effusion had lung cancer. Fifth, since our study involves hospitalized patients, our patients may have been sicker than patients who would be managed in an outpatient setting. Sicker patients may have higher serum LDH levels which could falsely elevate the cancer ratio. However, all of our patients hospitalized were stable despite pleural effusion. They were hospitalized predominantly to allow chest tube insertion and biopsy if need be as these require hospitalization in our setting for insurance claim purpose. A prospective study designed to overcome these limitations will help to validate our findings.

In conclusion, this is the first study to describe the ability to glean additional diagnostic information from a simple biomarker as serum LDH in pleural effusion. These findings can help in early (on first day of admission) identification of patients with malignant pleural effusion in a simple manner, with no added cost, or test. This may translate into the identification of patients for whom closed pleural biopsy may suffice (cancer ratio < 20) in view of its reasonable (70 %) diagnostic yield for TB, and those who may need thoracoscopic biopsy (cancer ratio >20) as the yield of closed pleural biopsy for diagnosing cancer is low. It may also find utility in predicting the frequency and duration of follow-up. Patients with an unconfirmed diagnosis (who refuse or are unfit for pleural biopsy) but who have a lower cancer ratio may be started on empirical TB treatment and may not require so frequent or prolonged follow-up with repeat chest imaging to assess for recurrence or interval worsening. In contrast, for patients with unconfirmed diagnosis but higher cancer ratio, it will identify the need for early follow-up and frequent or repeat chest imaging to assess for recurrence and early biopsy.
